# Lead-Free Piezoelectric Diaphragm Biosensors Based on Micro-Machining Technology and Chemical Solution Deposition

**DOI:** 10.3390/s16010069

**Published:** 2016-01-12

**Authors:** Xiaomeng Li, Xiaoqing Wu, Peng Shi, Zuo-Guang Ye

**Affiliations:** 1 Electronic Materials Research Laboratory, Key Laboratory of the Ministry of Education & International Center for Dielectric Research, Xi’an Jiaotong University, Xi’an 710049, China; leemoon56@stu.xjtu.edu.cn (X.L.); spxjy@mail.xjtu.edu.cn (P.S.); zye@sfu.ca (Z.-G.Y.); 2 Department of Chemistry and 4D LABS, Simon Fraser University, Burnaby, BC V5A 1S6, Canada

**Keywords:** piezoelectric diaphragm biosensor, lead-free, chemical solution deposition

## Abstract

In this paper, we present a new approach to the fabrication of integrated silicon-based piezoelectric diaphragm-type biosensors by using sodium potassium niobate-silver niobate (0.82KNN-0.18AN) composite lead-free thin film as the piezoelectric layer. The piezoelectric diaphragms were designed and fabricated by micro-machining technology and chemical solution deposition. The fabricated device was very sensitive to the mass changes caused by various targets attached on the surface of diaphragm. The measured mass sensitivity value was about 931 Hz/μg. Its good performance shows that the piezoelectric diaphragm biosensor can be used as a cost-effective platform for nucleic acid testing.

## 1. Introduction

Over the past few decades, researchers have focused on developing sensors with high sensitivity and low limits of detection. As a result, a wide range of biosensors with different operational modalities have been demonstrated. In particular, label-free biosensors are suitable for rapid and real-time sensing applications. Many types of label-free biosensors, such as nanogap and microneedle biosensors, have been widely studied [1,2,3,4,5,6,7,8].

In recent years, piezoelectric devices have been widely used as micro-pumps, actuators, transducers and biosensors [9,10,11,12,13,14,15,16,17,18]. The high sensitivity quartz crystal micro-balance (QCM) has been successfully used in biosensors for many years [19,20,21]. However, the conventional crystal or bulk ceramic technology is insufficient to meet the increasing requirements of miniaturization and integration. Recently, the development of micro-machined piezoelectric biosensors has become a focus of attention because of their merits such as compact size, high sensitivity, label-free operation, rapid response and compatibility with integrated circuit techniques [22,23,24,25,26,27]. Piezoelectric biosensors are mass-sensitive which is detected by measuring the shift of resonant frequency of the devices [25,28]. When the reaction between the recognition layer immobilized on the diaphragm and the captured target biological species happens, the total mass of the piezoelectric film area will change which results in a corresponding resonant frequency shift [29].

Nowadays, most piezoelectric devices use lead-based materials such as Pb(Zr, Ti)O_3_ (PZT) and Pb(Mg_1/3_Nb_2/3_)O_3_-PbTiO_3_ (PMN-PT) because of their excellent piezoelectric properties. However, the toxicity of lead has raised concerns and research on lead-free piezoelectric biosensors is in great demanded. The traditional lead-free piezoelectric materials, such as (K,Na)NbO_3_ (KNN) and (Bi,Na)TiO_3_ (BNT), are not widely applied because of their weak electrical properties. During this work, we have successfully prepared a new lead-free piezoelectric biosensor by using the lead-free film of 0.82K_0.5_Na_0.5_NbO_3_-0.18AgNbO_3_ (0.82KNN-0.18AN), which exhibited much improved electrical properties compared to pure KNN film [30].

## 2. Results and Discussion

### 2.1. Characterization of the 0.82KNN-0.18AN Piezoelectric Layer

**Figure 1:**
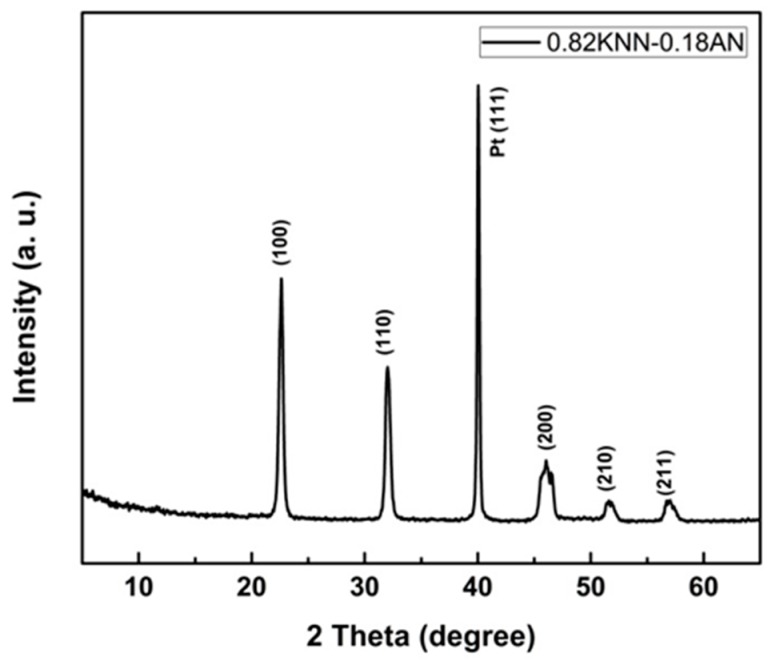
XRD pattern of the 0.82KNN-0.18AN film.

The crystallization and electrical properties of the 0.82KNN-0.18AN piezoelectric film were analysed. Figure 1 shows the XRD pattern of the 0.82KNN-0.18AN film, indicating that the film possessed a pure perovskite phase, and no obviously secondary phases were observed. It can be seen that the film exhibited (100) preferential orientation.

**Figure 2:**
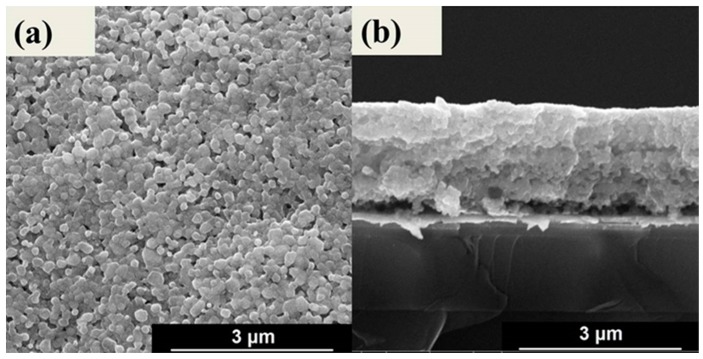
SEM images of the (**a**) surface; and (**b**) cross-sectional morphologies for the 0.82KNN-0.18AN film.

The surface and cross-sectional morphologies for the 0.82KNN-0.18AN film are shown in Figure 2. From the surface image, the average grain size of the 0.82KNN-0.18AN film was estimated to be about 200 nm. From the cross-sectional image, the 0.82KNN-0.18AN film thickness was calculated to be about 2.16 μm. The crack-free film showed a good structure which ensured the good quality of the biosensor.

**Figure 3:**
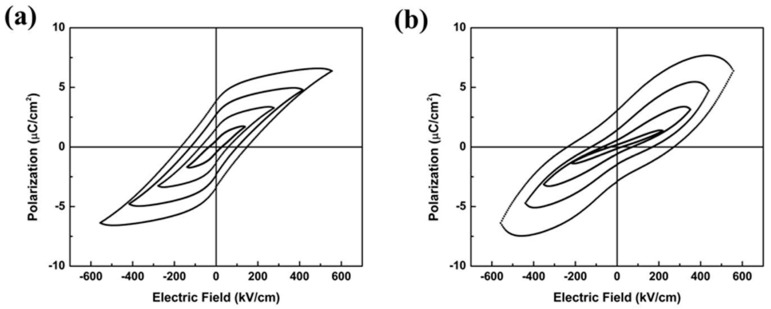
Hysteresis loops for the (**a**) 0.82KNN-0.18AN film; and (**b**) KNN film.

The 0.82KNN-0.18AN film showed well defined P-E hysteresis loops while the pure KNN film deposited with the same method showed poorly shaped P-E hysteresis loops, as demonstrated in Figure 3. The remnant polarization (Pr) of the 0.82KNN-0.18AN film was calculated as 3.59 μC/cm^2^, at the applied electric field of 550 kV/cm.

### 2.2. Characterization of the Piezoelectric Diaphragm Biosensor

**Figure 4:**
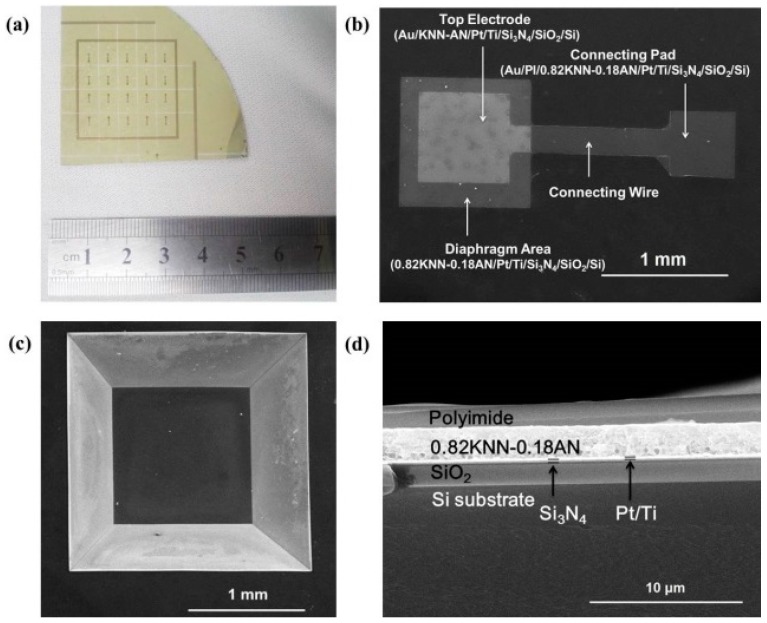
(**a**) Optical image; SEM images of the (**b**) front view; (**c**) back view; and (**d**) cross section of the fabricated biosensor.

Using the micro-machining process and deposition processing, piezoelectric diaphragm biosensors were successfully fabricated. As shown in Figure 4a, we can see that twenty individual biosensors were fabricated on a quarter of the 4” wafer. The SEM top and bottom views of one individual biosensor were shown in Figure 4b,c.

The structures of the diaphragm area, top electrode, connecting wire and connecting pad are shown in detail. The length of the biosensor was 1 mm and the length of the top electrode was 0.7 mm, which were the optimized parameters by the results of finite element simulation. The opening area of the chamber was much larger than that of the bottom diaphragm due to the anisotropic wet etching by KOH. The dipping and washing in the immobilization process were much more convenient due to this enlarged opening chamber. The cross section of the biosensor was shown in Figure 4d. Each layer of the multilayer structure was identified. The dense and crack-free 0.82KNN-0.18AN film ensured the high yield of the biosensor.

**Figure 5:**
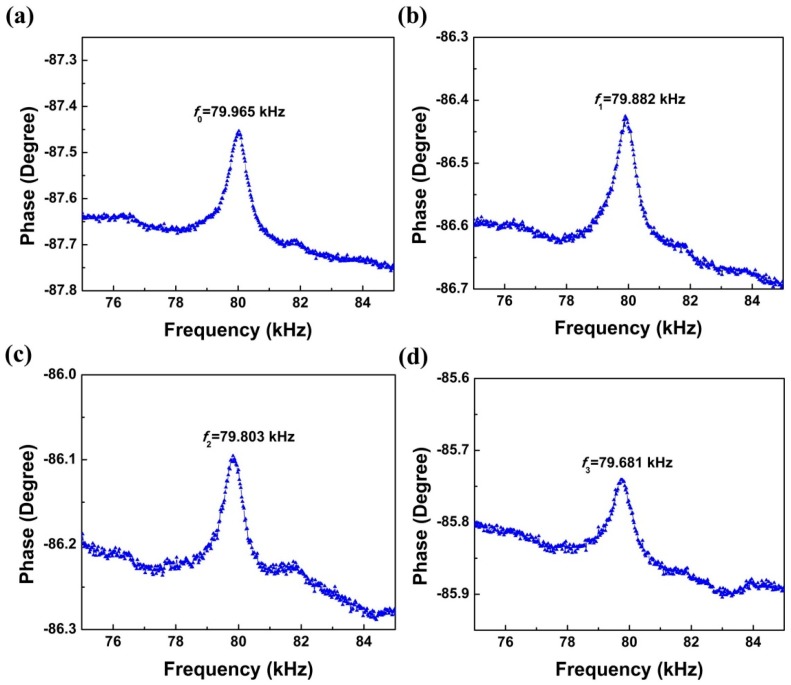
Resonant frequencies for different mass loaded: (**a**) no mass loaded; (**b**) 0.1 µg total mass loaded; (**c**) 0.2 µg total mass loaded and (**d**) 0.3 µg total mass loaded.

The test of mass sensitivity was carried out using a biosensor with a residual silicon thickness of about 25 μm. The detailed steps were as follows: firstly, we cleaned and modified the surface of a Au top electrode with piranha solution (70% H_2_SO_4_: 30% H_2_O_2_). Secondly, we added 0.1 µL of single-stranded DNA (ssDNA) (5′-6-FAM/CACAACAGACGGGCACACACTACT/C6-SH-3′) solution with a concentration of 1 µg/µL on the surface of top electrode via Au-S reaction as mass load. Thirdly, we dried the device at 37 °C for 30 min and hence 0.1 µg mass was loaded. The above adding-drying steps were repeated three times until a total of 0.3 µL of solution was added. The resonant frequencies were measured by an impedance analyzer (4294A, Agilent, Santa Clara, CA, USA) on the average resonant frequency values after three measurements. The relationship between resonant frequency and total mass load was thus obtained. The measured resonant frequencies were 79.965, 79.882, 79.803 and 79.681 kHz for 0, 0.1, 0.2, and 0.3 µg total loaded mass, respectively, as shown in Figure 5.

**Figure 6:**
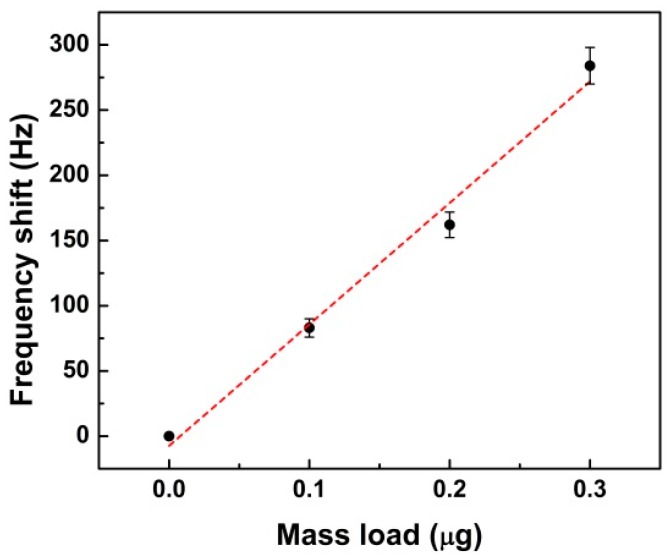
Relationship of the frequency shift and mass load.

A nearly linear relationship between the frequency shift and total mass load of the device was found, as shown in Figure 6. From the results, the mass sensitivity can be calculated as S_m_ = 931 Hz/μg. Xu *et al.* [25] reported a biosensor array for immunoassay with a mass sensitivity of 6250 Hz/μg. Their piezoelectric diaphragm biosensors were based on PZT films and had smaller dimensions, which could increase the mass sensitivity. Zhao *et al.* [29] reported a lead-free piezoelectric biosensor based on polyvinylidene fluoride (PVDF) piezoelectric film with mass sensitivity of 185 Hz/μg, which was much smaller than our result.

The bio-sensing performance characteristics were also studied. In this test, the residual silicon thickness of the biosensor was about 20 μm. The detailed steps were as follows: (1) Thin gold film of 50 nm-thick was deposited on the backside of the diaphragm to serve as the immobilization layer, which was then cleaned and modified by piranha solution treatment; (2) The capture DNA probe with the same concentration and sequence, which was used in the test of mass sensitivity, was immobilized on the gold surface. After 6 h of immobilization, the reaction surface was washed by DI water and dried under N_2_ flow; (3) 6-Mercapto-1-hexanol (MCH) blocking reagent was dropped for 2 h to improve the specificity. Then the reaction surface was washed by DI water and dried under N_2_ flow; (4) In the last step, target analytes which contain complementary DNA with the concentration of 1 µg/µL was added on the reaction surface for 0.5 h of nucleic acid hybridization. Then the excess analyte were washed away by DI water and the device was dried under N_2_ flow.

**Figure 7:**
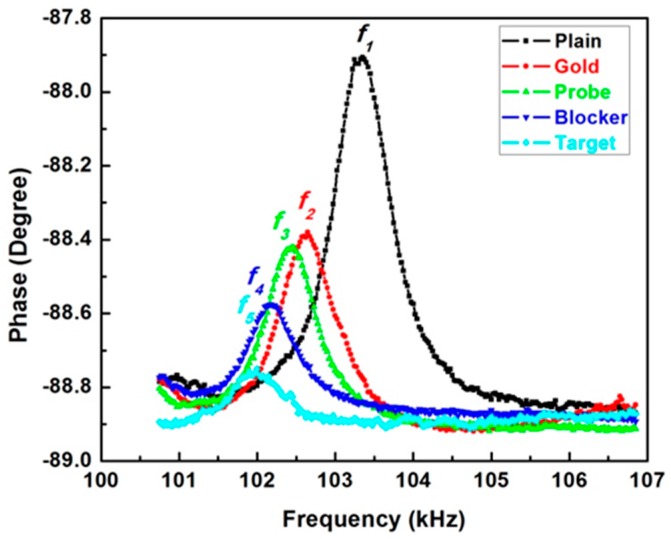
Frequency spectrum of the biosensor after different steps.

The resonant frequencies of the biosensor were measured immediately after each process, as shown in Figure 7. The original resonant frequency of the biosensor was noted as *f*_1_, and the resonant frequencies after depositing gold film, adding probes, blockers and targets are *f*_2_, *f*_3_, *f*_4_ and *f*_5_, respectively. The resonant frequency for the plain, the deposition of gold, the immobilization of DNA probes, blockers and targets are 103.341, 102.635, 102.427, 102.176 and 101.988 kHz, respectively. The resonant frequency was continuously shifted to the lower domain, indicating an accumulated mass increasing during each process.

**Figure 8:**
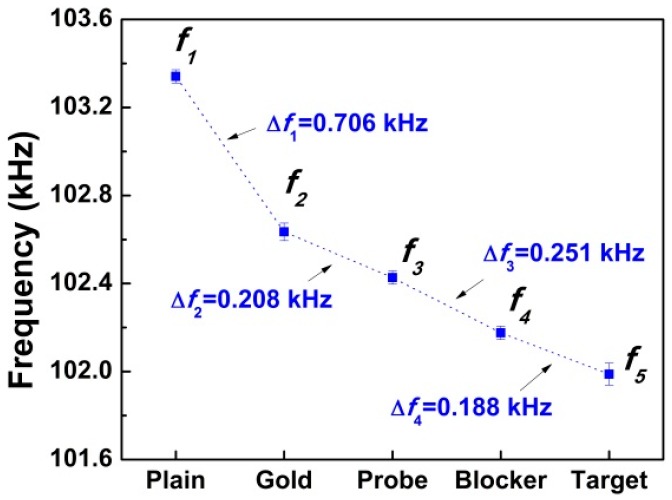
Frequency spectrum of the biosensor after different steps.

Detailed frequency changes after each process for the biosensor were shown in Figure 8, of which the data points were calculated based on the average values after three measurements. Comparing the frequency decreased values for the biosensor, ∆*f*_1_ = *f*_1_ − *f*_2_ is the largest, which may be due to the high density of gold film. The resonant frequency shift values for the immobilization of probes, blockers and targets are 0.208, 0.251 and 0.188 kHz, respectively.

## 3. Materials and Methods

### 3.1. Design of Device Structure and Sensing Principle

**Figure 9:**
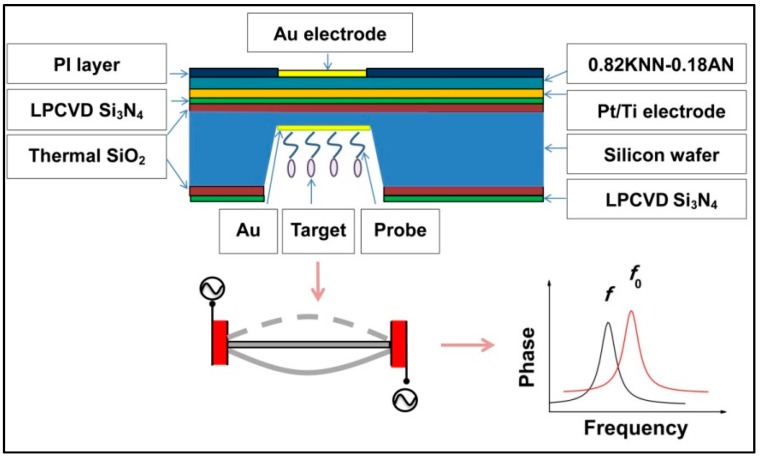
Schematic drawings and principle of the piezoelectric diaphragm biosensor.

The schematic structure and principle of the device are plotted in Figure 9. The piezoelectric biosensor was mainly composed of a supporting layer, piezoelectric layer, electrode layer and immobilization layer. For this piezoelectric diaphragm biosensor, the piezoelectric layer is driven by applying the voltage through the converse piezoelectric effect. The resonant frequency of biosensor after fabrication is *f*_0_, owing to a mass change of the diaphragm, the resonant frequency will decrease to *f*. The resonant frequency shift (*f* − *f*_0_) could be calculated, and thus the corresponding mass could be determined.

The resonant frequency of a square diaphragm with length of *a* can be expressed as [25,28,31,32]:

(1) $$f_{0}=\frac{1}{2\pi a^{2}}\sqrt{\frac{\gamma^{4}D}{\rho h}}$$

where *f*_0_ is the resonant frequency, ρ is the diaphragm density, *h* is the diaphragm thickness, γ is a constant and *D* is the flexural rigidity, respectively. The definition of mass sensitivity of the biosensor is the resonant frequency change corresponding to a unit mass load, which can be defined as [25,33]:

(2) $$S_{m}=-\frac{\Delta f}{\Delta m}$$

where ∆*m* is the mass change per unit area, the ∆*f* is the resonant frequency shift. A larger value of *S_m_* means that the biosensor is more mass sensitive.

### 3.2. Fabrication of the Piezoelectric Diaphragm Biosensor

**Figure 10:**
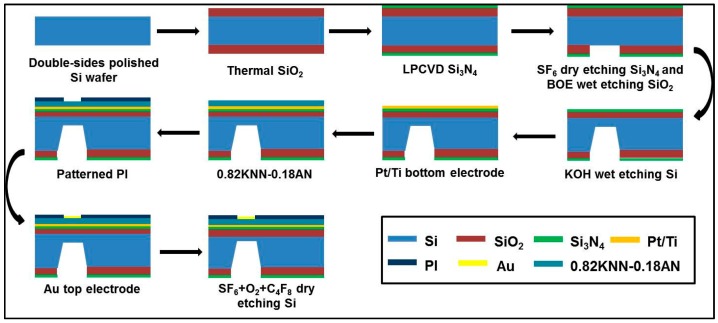
Schematic fabrication process of the piezoelectric diaphragms.

The piezoelectric diaphragms were fabricated by combining the chemical solution deposition method with traditional silicon micromachining technology; the fabrication processing steps are shown in Figure 10. In summary, the fabrication process was composed of nine main steps. (1) A layer of 1.5 μm-thick thermal SiO_2_ was grown on a 4” double-sided polished silicon wafer; (2) A silicon nitride layer of 200 nm was then deposited by low pressure chemical vapour deposition (LPCVD) on both sides of the wafer; (3) The diaphragm windows on the backside were opened by dry etching of Si_3_N_4_ and wet etching of SiO_2_, which were realized by inductive coupled plasma (ICP) using SF_6_ + He and by buffered oxide etchant (BOE, which was composed of HF, NH_4_F and H_2_O), respectively; (4) The backside silicon was anisotropic wet etched by KOH until the remaining thickness of silicon was about 50 μm; (5) Pt/Ti layer of 200 nm/20 nm were sputtered on the front side of wafer as the bottom electrode; (6) 0.82KNN-0.18AN layer was deposited using the chemical solution deposition technique to serve as the piezoelectric layer; (7) A polyimide (PI) layer of 2 μm was spin-coated, patterned and cured as an insulation layer to minimize parasitic capacitance induced by the patterned electrode wiring; (8) Au layer of 80 nm to serve as top electrode was sputtered and patterned by lift-off process; (9) Finally, the backside silicon was etched off by ICP using SF_6_ + O_2_ + C_4_F_8_ until the required residual silicon thickness (20~25 μm) was reached.

The thickness of the double-sided polished Si wafer was 400 μm and the crystal orientation was (100). Square diaphragms were fabricated by the anisotropic wet etching in 30 wt% KOH solution at 80 °C in the oil bath heater.

**Figure 11:**
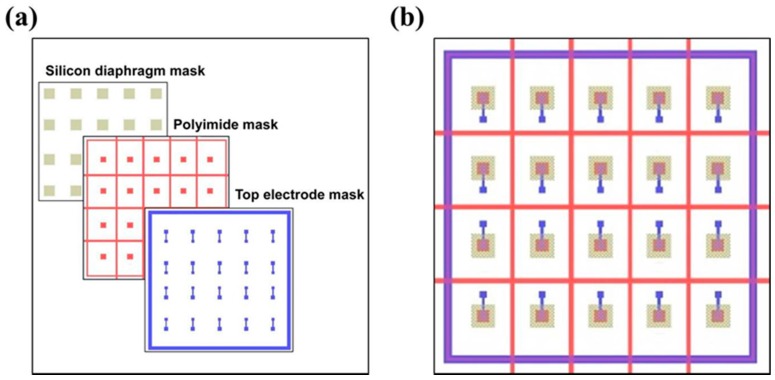
(**a**) Three masks for the fabrication process; and (**b**) their alignment image.

In the fabrication process, three lithography masks were used for the patterning of silicon diaphragms, the polyimide layer and the top electrodes, which were designed as shown in Figure 11. The alignment of the silicon diaphragm mask with the polyimide mask used the double-sided aligning technique while the polyimide mask with the top electrode mask used the overlay aligning technique.

The lead-free 0.82KNN-0.18AN thin film layer was deposited using the chemical solution deposition method. For the preparation of the 0.82KNN-0.18AN precursor solution, potassium acetate, sodium acetate, silver acetate and niobium ethoxide were used as the starting chemicals and 2-methoxyethanol was used as the solvent. Polyvinylpyrrolidone (PVP) with a molecular weight of 360,000 was added to enhance the film thickness. The chemical composition was designed as 0.82K_0.5_Na_0.5_NbO_3_-0.18AgNbO_3_. The final concentration of 0.82KNN-0.18AN precursor solution was 0.5 mol/L. The precursor solution was spin-coated onto the front side of the device at 2000 rpm for 60 s. Then wet film was dried at 100 °C for 2 min on a hotplate. The dried film was pyrolyzed at 330 °C for 5 min and annealed at 650 °C for 10 min in a rapid thermal annealing (RTA) furnace with a heating rate of 50 °C/s. Four layers were deposited by repeating all the above steps to increase the thickness of the 0.82KNN-0.18AN film.

### 3.3. Measurements of the Resonant Frequencies

The resonant frequencies were measured by the impedance analyzer (Agilent 4294A) at room temperature. The top electrode and bottom electrode were connected to the impedance analyzer using copper wires with a Agilent 42942A terminal adapter. During the measurements, the direct current (DC) bias voltage was fixed at 30 V and the oscillating (OSC) voltage level was 1 V.

## 4. Conclusions

In this paper, we have developed a novel lead-free piezoelectric diaphragm biosensor, unsing a fabrication process compatible with integrated circuit (IC) technology. The thickness of the piezoelectric film was about 2.16 μm. The resonant frequencies were measured and the frequency changes were calculated. The mass sensitivity of the biosensor was about 931 Hz/μg. After sensing performance characterization, it was verified that this piezoelectric biosensor can potentially be used for nucleic acid testing. With micro-machining technology and process optimization, the piezoelectric diaphragm biosensors can be miniaturized, so the cost can be further reduced.
